# 

**Published:** 1873-10

**Authors:** 


### Books and Pamphlets Received.

A Manual of Medical Jurisprudence. By Alfred Swaine Taylor, M. D., F. R. S. Seventh American Edition. Edited by John J. Reese, M. D. With Illustrations on wood. Philadelphia: Henry C. Lea, 1873. Buffalo: Theo. Butler & Son.

Chemistry, Inorganic and Organic, with Experiments. By Charles Loudon Bloxham, Professor of Chemistry in Kings College, London, &c. Philadelphia: Henry C. Lea, 1873. Buffalo: Theo. Butler & Son.

Handbook of Physiology. By William Senhouse Kirks, M. D. Edited by W. Morrant Baker, F. R. C. S. Philadelphia: Henry C. Lea, 1873. Buffalo: Theo. Butler & Son.

The London Medical Record. New York: G. P. Putnam & Sons, Buffalo: Martin Taylor.

Coccyodynia. A paper read before the Michigan State Medical Society. By Edward W. Jenks, M. D.

The Treatment of Typhoid Fever. By Jos. F. Montgomery, M. D., of Sacramento, Cal.

An Account of the Cholera as it appeared at Nashville, in the year 1873. By W. K. Bowling, M. D.

Transactions of the Medical and Chirurgical Faculty of the State of Maryland, April, 1873.

Law and Intelligence in Nature and the Improvement of the Race in accordance with Law. By A. B. Palmer, A. M., M. D.

The Therapeutic Effects and Uses of Mercury, as influenced by the Report of the Edinburgh Committee. By Wm. H. Doughty, M. D.

Transactions of California State Medical Society for the years 1872–73.

**Medical and Surgical Journal**

PUBLISHED MONTHLY,

**Containing Original Articles, Reports of Medical Societies and Hospitals, Editorial, Reviews, Correspondence, Army News, etc., etc.; including the usual variety of Medical Periodical Publications. Specimen copies sent on application.**

Address Buffalo Medical & Surgical Journal, Buffalo, N. Y.

**TERMS OF SUBSCRIPTION, - - $3.00 PER YEAR, IN ADVANCE.**

